# Research on the Characteristics and Usefulness of User Reviews of Online Mental Health Consultation Services: A Content Analysis

**DOI:** 10.3390/healthcare9091111

**Published:** 2021-08-27

**Authors:** Jingfang Liu, Lu Gao

**Affiliations:** School of Management, Shanghai University, Shanghai 201800, China; jingfangliu2014@hotmail.com

**Keywords:** online psychological consultation, online user reviews, review usefulness, topic analysis, sentiment analysis

## Abstract

Online consultation based on Internet technology is gradually becoming the main way to seek health information and professional assistance. Online user reviews, such as content reviews and star ratings, are an important basis for reflecting users’ views on the effectiveness of health services. Here, we used user reviews related to online psychological consultation services for content feature mining and usefulness analyses. We used a professional online psychological counseling service platform in China to collect user reviews that were liked by users as a data sample for a content analysis. An LDA topic model, dictionary-based sentiment analysis, and the NRC Word-Emotion Association Lexicon were used to extract the topic, sentiment, and context features of the content of 4254 useful reviews, and the influence of these features on the usefulness of the reviews was verified by a multiple linear regression analysis. Our results show that the content of online reviews by psychological counseling users presented a positive emotional attitude as a whole and expressed more views on the process, effects, and future expectations of counseling than on other topics. There was a significant correlation between the topic, sentiment, and context features of a user review and its usefulness: reviews giving high scores and containing topics such as “ease emotions” and “consulting expectations” received more user likes. However, the usefulness of a review was significantly reduced if it was in existence for too long. This research provides valuable suggestions for understanding the needs and emotional attitudes of users with mental health problems in terms of online psychological consultation; identifying the factors that affect the number of likes a review receives can help platform users write better consultation evaluations and thereby provide greater usefulness. In addition, the use of online reviews generated by users for content analysis effectively supplements the current research on online psychological counseling in terms of data and methods.

## 1. Introduction

### 1.1. Background

Online psychological consultation is a method of mental health consultation based on Internet technology (such as email, telephone, video, information, and forums) [1], and it is gradually becoming the main way for users with mental health problems to seek health information and professional assistance [2]. Studies have shown that online psychological counseling can provide a safe and exclusive emotional communication environment for users with mental illness [3] and can reduce time expenditures and space costs to a large extent [4]; thus, online psychological consulting is an effective online supplement to traditional face-to-face counseling in terms of improving timeliness and consistency [5]. For example, research by Weiss et al. on an online psychological counseling platform in Germany shows that online psychological counseling provides a promising way to help young people suffering from emotional crises [6]. However, due to a lack of trust in online health resources [7] and concerns about the uncertainty of the consultation process and effects [8,9], many users with mental illness fail to transform their willingness to engage in online psychological consultation services into actions [10].

According to research by Bird et al., the value of online counseling is an important predictor that affects users with mental illness who are seeking such help [11]. When they perceive that online counseling can provide valuable psychological support, the likelihood of these users using such services will be higher. For example, in research conducted by Baumel and Schueller, users believed that peer emotional support in an online psychological talking cure platform was more effective than drug therapy [12], so they were more willing to share their feelings and seek help on the platform. However, the current literature lacks research on the perceived value of the online counseling experience for users with mental illness. Although some scholars have employed interviews [13] or questionnaires [14] to investigate user satisfaction with utilizing online mental health resources to solve mental health problems, this research is limited by the research objects and insufficient coverage of the research data; moreover, the results, which consist of summarized topics and descriptive statistical analyses, lack a detailed and in-depth exploration of the effect of the user consultation experience.

Online user reviews contain a large amount of information about the effectiveness of counselors’ services, users’ own preferences, and the effects of users’ experiences [15], which helps other online platform users gain insights into the quality of services offered and the client–provider relationship [16] and influences their online decision-making in choosing a doctor [17,18]. Highly useful reviews are those that are more beneficial in providing an in-depth understanding of the real needs [19], implicit preferences [20], and information adoption [21] of users. For example, Salehan and Kim used emotion mining technology to construct a predictive model for the usefulness of online reviews and proved that neutral reviews have a positive impact on consumer purchasing decisions [22]. Similarly, our study of useful reviews on online psychological counseling platforms can help us more accurately analyze the counseling needs and feelings of visitors. This method is rarely mentioned in the current literature.

Therefore, the main purpose of this research was to conduct a content analysis of useful reviews, i.e., reviews from an online psychological consultation platform that were liked by users [23], specifically to analyze the key information and main characteristics of the content of these reviews by users with mental health problems through the topics and sentiments of the reviews and to determine how these review characteristics affect other users’ perceptions of the usefulness of the reviews on the platform.

### 1.2. Literature Review

#### 1.2.1. Online Psychological Consultation

In 2005, a review article by Mallen et al. on psychological counseling showed that online psychological counseling is considered a viable option for people who are often isolated [24]. In 1999, Grohol defined online psychological counseling as an information interaction process between help seekers and professionals that enables simultaneous or instant psychological counseling and treatment through the Internet. It is a new type of counseling mode that helps people solve life and relationship problems [25]. After that, scholars continued to explore and assess the attitudes and preferences of users with mental illness towards online counseling services by comparing these services with traditional face-to-face counseling services [26,27]. For example, in comparing face-to-face and telephone consultation methods, Hewitt et al. found that users usually use face-to-face communication to deal with multiple problems, while they use telephone consultation to address a single question [28]. After Lal and Adair observed in their 2014 literature review on electronic mental health that future research should give more attention to the suitability of online services, focusing on the needs of psychological counseling users and the greatest benefit potential [29], scholars gradually shifted their research focus to the needs and satisfaction of users [30] in order to improve the service level and utility value of online psychological consultation. For example, Serhal et al. measured the satisfaction of users with mental illness with five aspects of online health consultation services: access and timeliness, appropriateness, effectiveness, efficiency, and safety [31]. Thus, a comprehensive and in-depth understanding of the needs, attention, and emotional attitudes of users with mental illnesses regarding online counseling is an important factor in promoting the sustainable and healthy development of online psychological counseling services [11,32]. For privacy protection reasons, the identification of counselors and information about visitors’ consultations in online psychological counseling services cannot be made public. Therefore, user reviews have become important sources of information that reflect the consultation experiences and needs of users.

#### 1.2.2. Usefulness of User Reviews

Useful reviews can better reflect users’ focuses [33], and some users read useful reviews to decide whether to purchase a product or service [17]. Scholars Mudambi and Schuff defined the usefulness of online reviews as the perceived value of online reviews in the consumer decision-making process [34]. By sorting through the relevant literature on the usefulness of reviews, we found that the current research on the usefulness of online reviews is mainly concentrated in specific areas of the leisure and entertainment industry, such as retail platforms, restaurants, and hotels, while there are few studies on the medical service industry. However, online user reviews have been widely proven to provide valuable information and can help other users make medical decisions [35]. For example, a survey conducted in Germany showed that more than half of the visitors to an online platform would choose doctors based on online reviews and ratings [36]. Therefore, it is very important to ensure the reliability and usefulness of user reviews. Early research on the usefulness of reviews mainly focused on the score and source of reviews [37]. For example, a study by Forman et al. analyzed the impact of reviewers’ real names and geographic locations on the usefulness of their reviews [38], and Korfiatis et al. explained the relationship between ratings and review usefulness [39]. The research in recent years has deepened and given more attention to the impact of the implicit content and emotional characteristics of reviews on the value of reviews. For example, Lee et al. observed that scoring has limitations and provides a one-sided reflection of consumer opinions and that sentiment analysis is an effective way to improve reviews [40]. Table 1 shows the representative literature on the usefulness of reviews, including theoretical background, independent variables, dependent variables, and data sources.

The topic feature of a review’s content is the summary and generalization of the user’s experience and feelings regarding certain aspects of the product or service being reviewed and is an important factor that affects users’ adoption behavior [21]. For example, Hao and Zhang extracted hidden topics from online doctor reviews to examine Chinese users’ views of doctors and compare the differences between these topics in different medical professions [15]. Some scholars have used an LDA model to identify content topics as the influencing factors of users’ perceptions of the usefulness of reviews. Alodadi and Zhou extracted 20 topics from online doctor reviews, including doctors’ knowledge, doctors’ skills, and waiting time, and proved that combining topics and ratings can improve the usefulness of reviews [47]. Therefore, this research conducted review usefulness research by extracting topics from review content.

The sentiment characteristic of a review’s content is the description of its overall emotional tendency, which reflects the user’s intuitive emotional attitude towards the product or service being reviewed [48]. The topic feature is more reflective of the objective information content contained in a review, while the sentiment feature is more reflective of the user’s attitude and preference for the product or service from the perspective of perception. For example, Hu et al. analyzed review data related to Amazon book sales and found that there was an important relationship between the sentiment in review content and book sales [49]. The influence of sentiment characteristics on the usefulness of reviews has been proven in many studies. Salehan and Kim showed that reviews with positive sentimentality attracted more readers, while neutral reviews had a significant positive impact on consumers’ purchasing decisions [22]. Cao et al. observed that extreme emotional attitudes in review content can result in greater usefulness [50]. Therefore, this research conducted review usefulness research by extracting sentiment features from review content.

Based on the research status and previous research experience, this study took useful reviews of online mental health consultations as the research object and explored their content characteristics and how those characteristics affected other users’ perceptions of the usefulness of reviews from two perspectives: topics and sentiments.

## 2. Materials and Methods

### 2.1. Data Collection

The research for this article used secondary data. We collected user reviews and the related labels from a professional online psychological counseling service platform in China. This platform is one of China’s most popular Internet psychological counseling service platforms and a pioneer in the industry, with a wide audience and mature operating mechanism. Additionally, its data are used as a basis for academic research by many scholars [51].

There were two main reasons for choosing this online psychological counseling platform. On the one hand, the platform has strict rules for the admission of psychological counselors. For example, academic certificates and qualification certificates are displayed on the personal pages of psychological experts in the form of pictures, thereby ensuring the credibility and professionalism of the experts. Currently, more than 50% of the experts who provide services on the platform hold a bachelor’s, master’s, or doctoral degree in psychology. In addition, the platform has established close cooperative relations with hundreds of psychological institutions in China. On the other hand, the psychological counseling users on the platform can evaluate and provide feedback on the counselors’ services in the form of thank-you letters, and other users can respond with a “like”; the user reviews that have been liked are displayed in a separate “User Voice” module for platform users’ reference. Based on the above two reasons, the platform not only ensured the credibility of the source of the research data but also met the requirements of the research variables.

This study collected 33,024 review data points related to 312 consultants from February 2016 to December 2020. The specific data description is shown in Table 2. According to the research experience of previous scholars, the number of likes gained by a review is considered to be an effective indicator by which to measure the usefulness and reliability of the review [34]. Therefore, this study deleted the evaluations with 0 likes [52] and finally retained a total of 4254 reviews with likes as useful review content data for the research.

### 2.2. Topic Analysis

**Figure 1:**
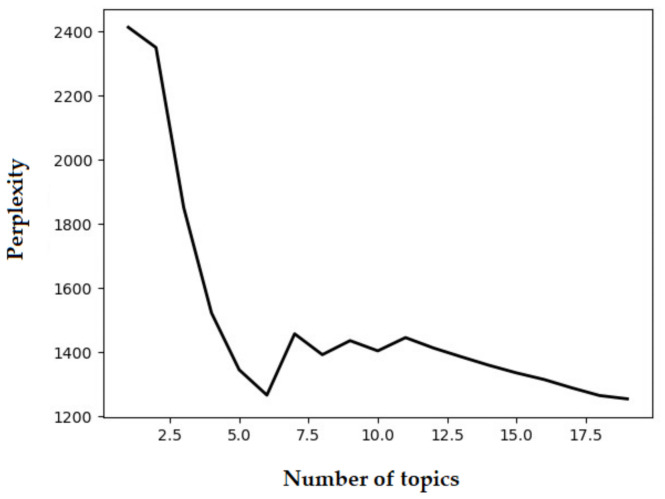
The perplexity of the topic model.

This study built an LDA topic model based on the Gensim program package in Python. LDA topic models are probabilistic models based on discrete data sets, such as probability data and a text corpus proposed by Blei [53]. Gensim is an open-source third-party Python toolkit that is used to learn without supervision the topic vector expression of the hidden layer of text from the original unstructured text. When using LDA for text mining, a perplexity indicator is used to determine the optimal number of topics. The smaller the perplexity value is, the greater the number of topics. In this study, the change in perplexity with the number of topics is shown in Figure 1. When K = 6, the perplexity of the LDA model was significantly smaller, and after that, the number of topics increased, and the degree of confusion gradually became flat. In addition, considering that too many topics would lead to too-small a granularity and overfitting of the model, the number of LDA topics was set to K = 6, and better training was obtained. When using the Gensim package to train the LDA model, the probability of each review belonging to a specific topic was stored in the variable LDA corpus, and the output result was used for the subsequent regression analysis.

### 2.3. Sentiment Analysis

The sentiment of a review includes two aspects: the sentiment score and sentiment polarity. The sentiment score was calculated using a dictionary-based method [54,55]. We searched for words of emotion in an emotional vocabulary, used each word of emotion as a benchmark, searched for adverbs of degree, and applied different weights according to the types of adverbs of degree to make corresponding score calculations, summed up the scores of each word of emotion in the review content, and, finally, processed the scores to prevent negative numbers from appearing. For emotional polarity, this study referred to the previous method and used the ratio of the number of positive emotion words to the number of negative emotion words as the measure [56]. The numbers of positive and negative emotion words were calculated by the NRC Word-Emotion Association Lexicon created by Mohammad and Turney [57]. The NRC Word-Emotion Association Lexicon is often used to perform fine-grained sentiment analyses on text [58]. However, since the original dictionary is in English, it was necessary to use Google Translate to translate the corresponding English into Chinese and then perform the calculations [57].

## 3. Results

### 3.1. Topic Analysis Results

Table 3 lists the extraction results related to the user review topics when K = 6. Selecting the top 10 keywords under each topic, we manually assigned a name to each topic to summarize the most likely content under the topic and calculated the overall probability distribution of each topic. The results show that the evaluation of online counseling by users with mental illness mainly involved six topics: gain change, ease emotions, clear thinking, patient listening, consulting expectations, and consulting feelings. Among them, “patient listening” had the highest proportion.

### 3.2. Sentiment Analysis Results

**Figure 2:**
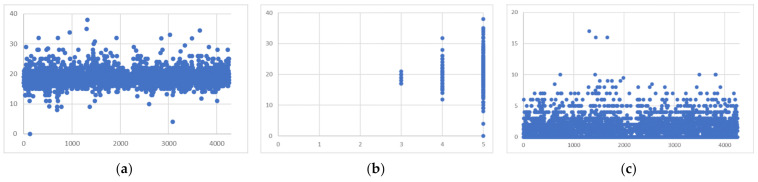
Sentiment analysis results: (**a**) Distribution of sentiment scores; (**b**) rating stars × emotional scores; (**c**) distribution of emotional polarity.

Figure 2 shows the results of sentiment analysis. The initial sentiment score was in the interval (−17, 21) and the final sentiment score after the nonnegative processing was in the interval (0, 38); that is, the boundary was 17, above which were the positive emotions and below which were the negative emotions. Figure 2a shows that the emotional scores were mainly concentrated in the 15–25 range, i.e., neutral emotional tendencies. Furthermore, we observed consistency between the review star ratings and the sentiment scores of the review content (Figure 2b). The number of stars, 1–5, represented ratings of very dissatisfied, relatively dissatisfied, average, relatively satisfied, or very satisfied. The neutral evaluation of 3 stars basically corresponded to review content with a neutral sentiment score. For the relatively satisfactory rating of 4 stars, many of the emotional scores were higher than 17; that is, they were in the range of positive emotions—only a small number of negative emotions appeared. For the 5-star very satisfactory evaluation, many of the emotional scores were relatively high. However, there were also obvious and extremely low emotional scores. Overall, the rating stars were basically consistent with the review sentiment. Figure 2c shows the distribution of sentiment polarity. We observed that the overall sentiment polarity of the reviews was positive and concentrated in the lower extreme of the value attributes.

### 3.3. Variable Descriptive Statistical Analysis

Table 4 presents a descriptive statistical analysis of the relevant variables in the regression analysis. According to previous research experience, the dependent variable used the number of likes to indicate the support of other users for the user reviews—that is, the perceived usefulness of the reviews [23]. The independent variables included five topic features (the sixth topic was removed due to multicollinearity) and emotional features including emotional scores and emotional polarity. To ensure the comprehensiveness and reliability of the regression analysis results, we added the review ratings and age as contextual features of the review content. Based on previous scholars’ research, such as Alodadi and Zhou, rating is a more significant factor affecting whether the review of a doctor is liked [47]; Lee proved that the age of a review is related to its usefulness [59]. Finally, because the research in this study mainly considered the usefulness factors from the perspective of user review content, external factors such as service price and quantity were used as control variables to avoid affecting the significance of the results.

### 3.4. Regression Analysis Results

A multiple regression analysis was performed to examine the impact of the review content features, including topic, sentiment, and context, on the usefulness of the dependent variable reviews. Table 5 shows the final regression results. We further evaluated the variance inflation factor of each independent variable (VIF) to test whether multicollinearity was a concern. The VIF shown in Table 5 is less than 5.0, which shows that multicollinearity was not a serious problem in the regression analysis in this study.

Model 1 examined the impact of the contextual features of a review’s content on the usefulness of the review. The regression results show that the rating level had a positive and significant impact on review usefulness, and review age was negatively significant. This shows that higher-scoring reviews are considered helpful and that the perceived usefulness of older reviews is significantly reduced. This finding is partly consistent with the research results of other scholars. The study by Hong et al. indicated that the age of a review has a significant impact on the usefulness of the review, while the rating level has no significant impact [23]. Our results show that in user reviews of online psychological consultations, the contextual characteristics of the review content, including ratings and age, significantly affected users’ perceptions of the usefulness of the reviews; the degree of influence of the ratings was higher.

Model 2 added the impact of the topic characteristics of the review content on the usefulness of the reviews on the basis of model 1. The results show that the five topics were positively and significantly related to the usefulness of the reviews. Among them, topic 1, “gain change”, had a significant positive correlation at the 0.05 level, while topics 2, 3, 4, and 5 were significantly positively correlated at the 0.01 level. This shows that the topic of a review is an important factor affecting the usefulness of the review. Topic 2, “ease emotions”, was a more significant factor in terms of increased usefulness, which is inconsistent with the findings of Alodadi and Zhou that “rating” is a more significant factor affecting the usefulness of reviews [47]. In addition, the topics of “patient listening” and “consulting expectations” were not only the most referenced topics in the user reviews but also had a relatively more obvious impact on users’ perceptions of the usefulness of the reviews. Compared with model 1, adding the topic feature of the review content improved model 2’s explanatory ability.

Model 3 added the influence of the emotional characteristics of the review content on the usefulness of the reviews on the basis of model 2. The results show that the emotional score and emotional polarity both positively affected the usefulness of the reviews at the 0.01 level. This shows that users expressing positive emotions or extreme positive emotions in reviews obtained more likes from other users. The early literature proposed the positive impact of positive emotions [22,33], but there have also been studies that have proven the role of negative emotions. For example, Laczniak et al. explained that consumers’ responses to negative reviews indicate that the spread of negative emotions from reviewers can enhance brand awareness [37]. However, in the online psychological consultation research scenario of this article, positive review sentiment was obviously more valuable for the usefulness of reviews. Compared with models 1 and 2, adding the emotional features of the review content further improved model 3’s interpretive ability.

## 4. Discussion

### 4.1. Principal Findings

Although online psychological counseling can provide more convenient and diverse mental health assistance services than traditional face-to-face counseling, we have a limited understanding of the online counseling experience and of the concerns of psychological counseling users. Given that online user reviews contain a wealth of information, it is not clear which review content features attract more users’ attention or are perceived to be more useful, thereby increasing the chances of successful psychological counseling matches. In the research for this article, we analyzed the content of useful reviews to prove that the topic “sentiment” and context characteristics of review content are important factors that affect the usefulness of user reviews of online psychological consultation services and made a contribution to the related research on online psychological consultation. This study was a new attempt to use online user reviews to mine users’ goals and emotional attitudes regarding online psychological counseling, and it is an effective supplement to the data and methods of current related research. The research results provide an in-depth understanding of the counseling needs and decision-making behaviors of users with mental health problems and can provide psychological counselors and platform managers with some suggestions for promoting users’ willingness to engage in online counseling.

The main findings are as follows:

1. The topics of user reviews of online mental health consultation services mainly involve descriptions of the consultation process and consultation effects, as well as future consultation expectations, which are significantly related to the increased usefulness of reviews. Among them, topics related to “ease emotions” and “consulting expectations” are more significant factors in improving the usefulness of reviews.

In the process of seeking online assistance for psychological counseling, whether users feel relieved and enlightened has the greatest impact on their perceptions of the value of counseling. For people seeking help with their health, the effect of treatment is a more direct and effective measure. The better the curative effect of a health service, the more users can perceive the usefulness of the health service. In addition, users with mental health problems state their thoughts and hopes for future consultations in reviews, and this type of content is also of concern to and recognized by other users. This is because a user’s expectations and feedback show gaps between the assistance provided by a consultant and the user’s true psychological needs and therefore can arouse empathy among other users.

2. The sentiment scores of user reviews of online mental health consultation services show a clear positive tendency and are consistent with ratings. Sentiment polarity is concentrated in the lower positive attributes, and there is a significant positive correlation between these sentiment features and the usefulness of reviews.

The overall emotional attitude of users with mental health problems towards online counseling is relatively optimistic and positive, which to a certain extent reflects that online psychological counseling has gradually become an effective way to provide psychological assistance; users’ acceptance of and satisfaction with it have continued to increase. In addition, positive emotional expressions in reviews result in more user likes. For users seeking professional psychological help, positive reviews can increase their trust in online consultation and their confidence in its effectiveness. In addition, users with mental illnesses tend to have negative emotions, so they also hope to ease their complex emotions through the positive emotions expressed by users who have received counseling services.

3. The contextual characteristics of user reviews of online mental health consultation services, including ratings and age, significantly affect users’ perceptions of the usefulness of reviews. Among them, the rating level is significantly correlated with a positive perception, and the age of a review is significantly correlated with a negative perception.

Review rating is a more intuitive indicator with which to judge user satisfaction. Consistent with emotions, higher ratings reflect users’ positive attitudes towards online psychological counseling services, thus greatly improving users’ perceptions of the usefulness of reviews. In addition, because reviews are time-sensitive, newly released reviews are easier for other users to view, are more representative, and have greater reference value, so they will receive more likes and support.

### 4.2. Theoretical Contributions and Practical Guidance

Theoretically, we emphasized the effects of the topic and sentiment aspects of user reviews, as well as the contextual conditions in which they exist, on the usefulness of user reviews. Before this, Alodadi and Zhou were the scholars to study the usefulness of user reviews, but they only considered the topic and sentiment aspects [47]. In addition, the research in this article supplements the current research in the context of online mental health counseling services, and the research results show that topics, sentiments, and contextual characteristics will have different effects on the usefulness of reviews by users with mental health problems.

In terms of practical guidance, our research results have certain reference significance for users and managers of online health community, especially psychological consulting service platforms. The results should improve the ability of psychological counselors to provide guidance. Patient listening, understanding, and acceptance are more conducive to attracting visitors than passing on professional knowledge. In addition, we should give attention to the consultation expectations of users, effectively meet the needs of users, and improve service satisfaction. Regarding platform manager actions, user reviews should be standardized to improve their reference value, and review recommendation mechanisms should be optimized so that other users with psychological counseling needs can obtain valuable input from users who have used online counseling services. For example, a unified review frame can be designed for users to fill in information relating to different topics. In addition, platforms can add “expert replies” to their review function to connect user feedback with expert replies. On the one hand, this can support timely communication between doctors and users after services terminate and can promote the establishment of a consultation relationship; on the other hand, it can also provide potential consultation users with richer knowledge of consultation experiences and references for doctor selection.

### 4.3. Limitations and Future Directions

Despite its contributions, this article also has some limitations. First, we only included reviews with likes in the data sample. Therefore, future research could take into account reviews that have not received likes for a comparative analysis and could thus expand the scope of the data to reveal new information. Second, we only considered the textual characteristics of the reviews as the independent variable. If we had taken reviewer and consultant information into account—such as gender, age, type and duration of consultation, and treatment method—we may have had different findings. Finally, we mainly analyzed users’ attitudes towards the effectiveness of e-health based on online reviews. Further research should consider other measurements, such as satisfaction questionnaires and health questionnaires, before and after the intervention, so as to expand the research data and confirm the research results.

## 5. Conclusions

In general, there are few studies on the characteristics and usefulness of user reviews of online mental health consultations. This article analyzed content characteristics of the reviews of psychological counseling users in detail and how these characteristics affected the usefulness of the reviews in determining the users’ counseling experiences and needs. The results of this study show that the content of online reviews by psychological counseling users presented a positive emotional attitude as a whole and expressed more views on the counseling process, counseling effects, and future expectations than on other topics. The topic, sentiment, and context of a review are significantly related to the usefulness of the review. Reviews with high ratings and content containing the topic “ease emotions” obtain more user likes, but the usefulness of older reviews is significantly reduced. The results of the research can help provide an in-depth understanding of the needs and concerns of psychological counseling users and can have significance as a reference in improving the online service level of counselors and standardizing the management of user reviews on online platforms.

## Figures and Tables

**Table 1:** Literature review on the usefulness of reviews.

Author	Theoretical Background	IV	DV	Data Source
Mudambi and Schuff (2010) [34]	Information economics theory	Review extremity Review depth	Review helpfulness	Amazon.com
Baek et al. (2014) [41]	Dual process theories	Rating inconsistency Reviewer credibility Word count Negative word	Review helpfulness	Amazon.com
Yin et al. (2014) [42]	Theories of emotion	Emotions (anxiety; anger)	Review helpfulness	---
Hu and Chen (2016) [43]	Review content analysis Review polarity	Review content Review sentiment Review author Review visibility	Review helpfulness	TripAdvisor.com
Zhou et al. (2020) [44]	Mere exposure theory	Title-content similarity	Review helpfulness	Amazon.com
Chen and Farn (2020) [45]	Attribution theory Cognitive appraisal theory	Negative emotions Positive emotions	Review helpfulness	---
Evans et al. (2021) [46]	Expressions of doubt and trust	Expressions of doubt	Review helpfulness	Yelp

Notes: IV: independent variables; DV: dependent variables.

**Table 2:** Data description.

Pro	Quantity	Percent
0	28,769	87.12%
1	2834	8.58%
2	463	1.40%
3	207	0.63%
4	105	0.32%
5	70	0.21%
6–10	206	0.62%
11–15	175	0.53%
16–20	152	0.46%
21–27	43	0.13%
Sum	33,024	100%

**Table 3:** Topic analysis results.

Topic 1	Topic 2	Topic 3	Topic 4	Topic 5	Topic 6
gain	feel	thinking	patient	expect	satisfied
method	emotions	ponder	comfortable	feel	thanks
kids	unblock	clear	professional	first time	feeling
grow up	self	relationship	analysis	hope	very good
change	understand	guide	listen	next time	meticulous
suggest	inner heart	cognition	help	help	objective
face	accept	idea	talk	resolve	great
life	walk out	explore	communicate	heart	warm
confidence	fear	define	voice	truly	gentle
love	subconscious	value	warm	demand	happy
gain change 13.83%	ease emotions 13.44%	clear thinking 14.60%	patient listening 28.54%	consulting expectations 17.66%	consulting feelings 11.93%

**Table 4:** Descriptive statistical analysis of variables.

Variables	Description	Mean	SD	Min	Max
***Dependent variable***					
Pro	Number of likes from other users	2.983	4.486	1	27
***Topic features***					
Topic 1	Consultation effect and related topics	0.138	0.178	0.0149	0.857
Topic 2	Emotional acceptance and related topics	0.134	0.172	0.0149	0.902
Topic 3	Clear thinking and related topics	0.146	0.192	0.0174	0.889
Topic 4	Listening patiently and related topics	0.285	0.268	0.0150	0.889
Topic 5	Consultation expectations and related topics	0.177	0.213	0.0159	0.893
***Sentiment features***					
Scores	Sentiment score calculated by sentiment dictionary	18.52	2.269	0	38
Polarity	Ratio of positive emotions to negative emotions	1.339	1.591	0	17
***Contextual features***					
Rating	Review rating (1–5)	4.934	0.259	3	5
Age	Difference between review time and data collection time	411.5	313.0	0	1730
***Control variables***					
Price	Consulting service price	483.4	163.8	200	1700
Volume	Total number of consulting services	1567	1577	9	8161

Notes: SD = standard deviation, Min = minimum, Max = maximum.

**Table 5:** Regression analysis results.

Variables	Pro					
	Model 1	Model 2	Model 3	95% Confidence Intervals		VIF
Price	0.002 ***	0.002 ***	0.002 ***	0.001	0.003	1.18
	(0.000) *t* = 4.18	(0.000) *t* = 4.12	(0.000) *t* = 3.86			
Volume	0.000 ***	−0.000 ***	−0.000 ***	−0.001	−0.000	1.19
	(0.000) *t* = −7.32	(0.000) *t* = −7.77	(0.000) *t* = −7.39			
Rating	1.054 ***	1.074 ***	1.005 ***	0.505	1.505	1.00
	(0.258) *t* = 4.09	(0.257) *t* = 4.18	(0.255) *t* = 3.94			
Age	−0.003 ***	−0.003 ***	−0.003 ***	−0.003	−0.002	1.01
	(0.000) *t* = −13.75	(0.000) *t* = −13.62	(0.000) *t* = −14.28			
Topic 1		1.422 **	1.086 *	0.070	2.102	1.96
		(0.521) *t* = 2.73	(0.518) *t* = 2.10			
Topic 2		2.571 ***	2.019 ***	0.984	3.054	1.90
		(0.528) *t* = 4.87	(0.528) *t* = 3.82			
Topic 3		1.735 ***	1.348 **	0.371	2.325	2.10
		(0.500) *t* = 3.47	(0.498) *t* = 2.71			
Topic 4		2.234 ***	1.677 ***	0.834	2.521	3.05
		(0.429) *t* = 5.21	(0.430) *t* = 3.90			
Topic 5		2.384 ***	1.943 ***	1.012	2.874	2.35
		(0.476) *t* = 5.01	(0.475) *t* = 4.09			
Scores			0.110 ***	0.049	0.172	1.15
			(0.031) *t* = 3.55			
Polarity			0.279 ***	0.192	0.366	1.16
			(0.045) *t* = 6.26			
Observations	4.254	4.254	4.254			
*R* ^2^	0.060	0.068	0.084			
Adjusted *R*^2^	0.0587	0.0656	0.0814			
*F*	67.35	34.17	35.25			

Notes: Significance: *** *p* < 0.001, ** *p* < 0.01, * *p* < 0.05. The first entry in the table is the beta weight. The numbers in brackets are the standard errors. The third entry is the *t*-value. *R*^2^ values: 0.25, 0.50, and 0.75 indicate weak, moderate, and substantial predictive power [60]. The 95% confidence intervals for the regression coefficients of model 3 are presented. In order to eliminate multicollinearity, we calculated the VIF value.

## References

[1] Barak A., Klein B., Proudfoot J.G. (2009). Defining internet-supported therapeutic interventions. Ann. Behav. Med..

[2] Ramaswamy A., Yu M., Drangsholt S., Ng E., Culligan P.J., Schlegel P.N., Hu J.C. (2020). Patient Satisfaction With Telemedicine During the COVID-19 Pandemic: Retrospective Cohort Study. J. Med. Internet Res..

[3] King R., Bambling M., Lloyd C., Gomurra R., Smith S., Reid W., Wegner K. (2006). Online counselling: The motives and experiences of young people who choose the Internet instead of face to face or telephone counselling. Couns. Psychother. Res..

[4] Chan J.K., Farrer L.M., Gulliver A., Bennett K., Griffiths K.M. (2016). University Students’ Views on the Perceived Benefits and Drawbacks of Seeking Help for Mental Health Problems on the Internet: A Qualitative Study. JMIR Hum. Factors.

[5] Paul C.L., Boyes A.W., O’Brien L., Baker A.L., Henskens F.A., Roos I., Clinton-McHarg T., Bellamy D., Colburn G., Rose S. (2016). Protocol for a Randomized Controlled Trial of Proactive Web-Based Versus Telephone-Based Information and Support: Can Electronic Platforms Deliver Effective Care for Lung Cancer Patients?. JMIR Res. Protoc..

[6] Weiss M., Hildebrand A., Braun-Scharm H., Weckwerth K., Held D., Stemmler M. (2020). Are suicidal young people reached by online-counselling? Evaluation of the target group outreach of [U25] online suicide prevention. Z. Kinder Jugendpsychiatrie Psychother..

[7] Huang H., Yan X., Yu L., Yu Z. (2013). Relation of attitude towards online counseling to personality traits andinternet self-efficacy in college students. Chin. Ment. Health J..

[8] McMillan S.J., Hwang J.S. (2002). Measures of perceived interactivity: An exploration of the role of direction of communication, user control, and time in shaping perceptions of interactivity. J. Advert..

[9] Mayer G., Gronewold N., Alvarez S., Bruns B., Hilbel T., Schultz J.H. (2019). Acceptance and Expectations of Medical Experts, Students, and Patients Toward Electronic Mental Health Apps: Cross-Sectional Quantitative and Qualitative Survey Study. JMIR Ment. Health.

[10] March S., Day J., Ritchie G., Rowe A., Gough J., Hall T., Yuen C.Y.J., Donovan C.L., Ireland M. (2018). Attitudes Toward e-Mental Health Services in a Community Sample of Adults: Online Survey. J. Med. Internet Res..

[11] Bird M.D., Chow G.M., Yang Y. (2020). College students’ attitudes, stigma, and intentions toward seeking online and face-to-face counseling. J. Clin. Psychol..

[12] Baumel A., Schueller S.M. (2016). Adjusting an Available Online Peer Support Platform in a Program to Supplement the Treatment of Perinatal Depression and Anxiety. JMIR Ment. Health.

[13] Navarro P., Bambling M., Sheffield J., Edirippulige S. (2019). Exploring Young People’s Perceptions of the Effectiveness of Text-Based Online Counseling: Mixed Methods Pilot Study. JMIR Ment. Health.

[14] Pretorius C., Chambers D., Cowan B., Coyle D. (2019). Young People Seeking Help Online for Mental Health: Cross-Sectional Survey Study. JMIR Ment. Health.

[15] Hao H., Zhang K. (2016). The Voice of Chinese Health Consumers: A Text Mining Approach to Web-Based Physician Reviews. J. Med. Internet Res..

[16] Hong Y.A., Liang C., Radcliff T.A., Wigfall L.T., Street R.L. (2019). What Do Patients Say About Doctors Online? A Systematic Review of Studies on Patient Online Reviews. J. Med. Internet Res..

[17] Ghose A., Ipeirotis P.G. Designing novel review ranking systems: Predicting the usefulness and impact of reviews. Proceedings of the 9th International Conference on Electronic Commerce.

[18] Zhao K., Yang X., Tao X., Xu X., Zhao J. (2020). Exploring the Differential Effects of Online Reviews on Film’s Box-Office Success: Source Identity and Brand Equity From an Integrated Perspective. Front. Psychol..

[19] Fang B., Ye Q., Kucukusta D., Law R. (2016). Analysis of the perceived value of online tourism reviews: Influence of readability and reviewer characteristics. Tour. Manag..

[20] Chen S., Lv X., Gou J. (2020). Personalized Recommendation Model: An Online Comment Sentiment Based Analysis. Int. J. Comput. Commun..

[21] Paulsell D., Thomas J., Monahan S., Seftor N.S. (2017). A Trusted Source of Information: How Systematic Reviews Can Support User Decisions About Adopting Evidence-Based Programs. Eval. Rev..

[22] Salehan M., Kim D.J. (2016). Predicting the performance of online consumer reviews: A sentiment mining approach to big data analytics. Decis. Support Syst..

[23] Hong H., Xu D., Wang G.A., Fan W.G. (2017). Understanding the determinants of online review helpfulness: A meta-analytic investigation. Decis. Support Syst..

[24] Mallen M.J., Vogel D.L., Rochlen A.B., Day S.X. (2005). Online counseling: Reviewing the literature from a counseling psychology framework. Couns. Psychol..

[25] Grohol J.M. (1999). The Insider’s Guide to Mental Health Resources Online. Cyberpsychol. Behav..

[26] Wetterlin F.M., Mar M.Y., Neilson E.K., Werker G.R., Krausz M. (2014). eMental health experiences and expectations: A survey of youths’ Web-based resource preferences in Canada. J. Med. Internet Res..

[27] Kauer S.D., Mangan C., Sanci L. (2014). Do online mental health services improve help-seeking for young people? A systematic review. J. Med. Internet Res..

[28] Hewitt H., Gafaranga J., McKinstry B. (2010). Comparison of face-to-face and telephone consultations in primary care: Qualitative analysis. Br. J. Gen. Pract..

[29] Lal S., Adair C.E. (2014). E-mental health: A rapid review of the literature. Psychiatr. Serv..

[30] Im S., Lee J., Han S. (2017). Video-counseling: Needs Assessment and Perception of Service Utilization. Korean J. Stress Res..

[31] Serhal E., Kirvan A., Sanches M., Crawford A. (2020). Client Satisfaction and Experience With Telepsychiatry: Development and Validation of a Survey Using Clinical Quality Domains. J. Med. Internet Res..

[32] Chan M., Li X.Q. (2020). Smartphones and psychological well-being in China: Examining direct and indirect relationships through social support and relationship satisfaction. Telemat. Inform..

[33] Schindler R.M., Bickart B. (2012). Perceived helpfulness of online consumer reviews: The role of message content and style. J. Consum. Behav..

[34] Mudambi S.M., Schuff D. (2010). What Makes a Helpful Online Review? A Study of Customer Reviews on Amazon.com. MIS Q..

[35] Hao H. (2015). The development of online doctor reviews in China: An analysis of the largest online doctor review website in China. J. Med. Internet Res..

[36] Ghose A., Ipeirotis P.G. (2011). Estimating the Helpfulness and Economic Impact of Product Reviews: Mining Text and Reviewer Characteristics. IEEE Trans. Knowl. Data Eng..

[37] Laczniak R.N., DeCarlo T.E., Ramaswami S.N. (2001). Consumers’ Responses to Negative Word-of-Mouth Communication: An Attribution Theory Perspective. J. Consum. Psychol..

[38] Forman C., Ghose A., Wiesenfeld B. (2008). Examining the relationship between reviews and sales: The role of reviewer identity disclosure in electronic markets. Inform. Syst. Res..

[39] Korfiatis N., Garcia-Bariocanal E., Sanchez-Alonso S. (2012). Evaluating content quality and helpfulness of online product reviews: The interplay of review helpfulness vs. review content. Electron. Commer. Res. Appl..

[40] Lee S.W., Jiang G.B., Kong H.Y., Liu C. (2020). A difference of multimedia consumer’s rating and review through sentiment analysis. Multimed. Tools Appl..

[41] Baek H., Ahn J., Choi Y. (2014). Helpfulness of Online Consumer Reviews: Readers’ Objectives and Review Cues. Int. J. Electron. Commer..

[42] Yin D., Bond S.D., Zhang H. (2014). Anxious or Angry? Effects of Discrete Emotions on the Perceived Helpfulness of Online Reviews. MIS Q..

[43] Hu Y.-H., Chen K. (2016). Predicting hotel review helpfulness: The impact of review visibility, and interaction between hotel stars and review ratings. Int. J. Inf. Manag..

[44] Zhou Y., Yang S., Li Y., Chen Y., Yao J., Qazi A. (2020). Does the review deserve more helpfulness when its title resembles the content? Locating helpful reviews by text mining. Inform. Process. Manag..

[45] Chen M.-J., Farn C.-K. (2020). Examining the Influence of Emotional Expressions in Online Consumer Reviews on Perceived Helpfulness. Inform. Process. Manag..

[46] Evans A.M., Stavrova O., Rosenbusch H. (2021). Expressions of doubt and trust in online user reviews. Comput. Hum. Behav..

[47] Alodadi N., Zhou L.N. Predicting the Helpfulness of Online Physician Reviews. Proceedings of the 2016 IEEE International Conference on Healthcare Informatics (ICHI).

[48] Berkovic D., Ackerman I.N., Briggs A.M., Ayton D. (2020). Tweets by People With Arthritis During the COVID-19 Pandemic: Content and Sentiment Analysis. J. Med. Internet Res..

[49] Hu N., Koh N.S., Reddy S.K. (2014). Ratings lead you to the product, reviews help you clinch it? The mediating role of online review sentiments on product sales. Decis. Support. Syst..

[50] Duan W., Cao Q., Gan Q. (2010). Investigating determinants of voting for the “helpfulness” of online consumer reviews: A text mining approach. AMCIS.

[51] Zhou J., Zuo M., Ye C. (2019). Understanding the factors influencing health professionals’ online voluntary behaviors: Evidence from YiXinLi, a Chinese online health community for mental health. Int. J. Med. Inform..

[52] Wang Y., Wang J., Yao T., Li M. (2020). What makes peer review helpfulness evaluation in online review communities? An empirical research based on persuasion effect. Online Inf. Rev..

[53] Blei D.M., Ng A.Y., Jordan M.I. (2003). Latent Dirichlet Allocation. J. Mach. Learn. Res..

[54] Xie R., Chu S.K.W., Chiu D.K.W., Wang Y. (2021). Exploring Public Response to COVID-19 on Weibo with LDA Topic Modeling and Sentiment Analysis. Data Inf. Manag..

[55] Pengnate S., Riggins F.J. (2020). The role of emotion in P2P microfinance funding: A sentiment analysis approach. Int. J. Inf. Manag..

[56] Geetha M., Singha P., Sinha S. (2017). Relationship between customer sentiment and online customer ratings for hotels—An empirical analysis. Tour. Manag..

[57] Mohammad S.M., Turney P.D. (2013). Crowdsourcing a Word-Emotion Association Lexicon. Comput. Intell..

[58] Malik M.S.I., Hussain A. (2017). Helpfulness of product reviews as a function of discrete positive and negative emotions. Comput. Hum. Behav..

[59] Lee J. (2013). What makes people read an online review? The relative effects of posting time and helpfulness on review readership. Cyberpsychol. Behav. Soc. Netw..

[60] Henseler J., Ringle C.M., Sinkovics R.R. (2009). The use of partial least squares path modeling in international marketing. Adv. Int. Mark..

